# Neuroinflammation induces glial aromatase expression in the uninjured songbird brain

**DOI:** 10.1186/1742-2094-8-81

**Published:** 2011-07-18

**Authors:** Kelli A Duncan, Colin J Saldanha

**Affiliations:** 1 Department of Biological Sciences, Lehigh University, 111 Research Dr., Bethlehem, Pennsylvania, 18015, USA

**Keywords:** Aromatase, Cytokine, Estrogen, Neuroinflammation, Glia

## Abstract

**Background:**

Estrogens from peripheral sources as well as central aromatization are neuroprotective in the vertebrate brain. Under normal conditions, aromatase is only expressed in neurons, however following anoxic/ischemic or mechanical brain injury; aromatase is also found in astroglia. This increased glial aromatization and the consequent estrogen synthesis is neuroprotective and may promote neuronal survival and repair. While the effects of estradiol on neuroprotection are well studied, what induces glial aromatase expression remains unknown.

**Methods:**

Adult male zebra finches (*Taeniopygia guttata*) were given a penetrating injury to the entopallium. At several timepoints later, expression of aromatase, IL-1β-like, and IL-6-like were examined using immunohisotchemistry. A second set of zebra birds were exposed to phytohemagglutinin (PHA), an inflammatory agent, directly on the dorsal surface of the telencephalon without creating a penetrating injury. Expression of aromatase, IL-1β-like, and IL-6-like were examined using both quantitative real-time polymerase chain reaction to examine mRNA expression and immunohistochemistry to determine cellular expression. Statistical significance was determined using t-test or one-way analysis of variance followed by the Tukey Kramers post hoc test.

**Results:**

Following injury in the zebra finch brain, cytokine expression occurs prior to aromatase expression. This temporal pattern suggests that cytokines may induce aromatase expression in the damaged zebra finch brain. Furthermore, evoking a neuroinflammatory response characterized by an increase in cytokine expression in the uninjured brain is sufficient to induce glial aromatase expression.

**Conclusions:**

These studies are among the first to examine a neuroinflammatory response in the songbird brain following mechanical brain injury and to describe a novel neuroimmune signal to initiate aromatase expression in glia.

## Background

Damage to the homeotherm brain increases aromatase (*estrogen synthase*) in reactive astroglia [1-3]. Although constitutive aromatase is abundant and neuronal in the undamaged songbird brain, glial aromatase expression is rapidly upregulated following brain damage [1,4-8]. Increased transcription and translation of glial aromatase occurs following damage to the neuropil in songbirds and to a lesser extent in mammals [2,8-10]. In songbirds, this upregulation appears more rapid and robust, since the secondary wave of degeneration characteristic of the mammalian (including human) brain following TBI is only revealed in songbirds following inhibition of upregulated glial aromatase [3,11]. Indeed, estrogen derived from glial aromatase may act by decreasing reactive gliosis that inhibits neurodegeneration [11]. Further, following injury, estrogens serve to limit further damage [3,9,10,12,13] by decreasing neurodegenerative properties and promoting neuroprotective pathways [7,14,15]. While much attention has been paid to the physiological mechanisms whereby estrogen mitigates damage and accelerates repair, virtually nothing is known about what is responsible for the induction of aromatase in astrocytes.

Among the many changes that accompany traumatic brain injury (TBI), neuroinflammation due to disruption of the blood brain barrier may provide a plausible signal to induce aromatase transcription in reactive astroglia [16-19]. TBI is characterized by both the physical damage and a secondary neuroinflammatory response characterized by increased cytokine and chemokine expression [19-22]. In very general terms, these events may be separated into two distinct, but interrelated phases. In the initial phase the mechanical injury creates a physical trauma to the brain that results in tissue damage and cell death [19,21,22]. The secondary phase of TBI is due to the disruption of the blood brain barrier (BBB) with a subsequent immune and inflammatory response [19,21,22]. These effects can occur within minutes of the trauma and last for weeks to even months later [22]. The neuroinflammatory response (characterized by increased cytokine expression) following injury can exert both neurotoxic (inflammation, brain swelling) and neuroprotective (promoting phagocytosis and repair) actions [18,23].

Cytokines (Interleukins, Tumor Necrosis factors, Transforming Growth Factors) like aromatase are also upregulated following injury or damage to the brain. Their presence following injury has implicated them as mediators and inhibitors of neurodegeneration [17,19,23-26]. Cytokine production is not only due to infiltrating immune cells but also from reactive astrocytes. Moreover, microinjections of cytokines into a rat stab wound significantly increase astrogliosis and cytokines have been implicated in regulating homeostasis in tissues and promoting repair following disease [19]. Furthermore cytokines regulate aromatase gene expression via alternate promoters in normal and malignant breast tissue [27-30] and may provide a plausible signal for the induction of glial aromatase. While IL-1 has some ability to increase aromatase activity, the most potent cytokine stimulator of aromatase is IL-6, a pro-inflammatory cytokine that is also upregulated following TBI [21,29-31]. These data suggest a unique and endogenous protective mechanism that reduces neurodegeneration and may actively inhibit the deleterious effects of prolonged cytokine action following brain damage.

Here we test the hypothesis that induction of a neuroinflammatory response independent of TBI is capable of inducing aromatase transcription/translation in zebra finch astrocytes. First, we tested whether neuroinflammatory cytokine expression (IL-1 & IL-6) precedes glial aromatase expression following brain injury. Next, we tested if an inflammatory response, independent of a penetrating injury, induces astrocytic glial aromatase.

## Methods

### Birds

Adult male zebra finches (> 90 days post-hatching) were obtained from a breeder (Magnolia Bird Farms; Anaheim, CA) and housed in the animal facility at Lehigh University. The Lehigh University Institutional Use and Animal Care Committee (IACUC) approved of all animal procedures.

Preliminary Studies. Verification of antigenic IL-1β, IL-6, and aromatase in zebra finches via Western Blotting.

The antibody used to detect aromatase has been extensively characterized [32,33]. To determine if antibodies against IL-1β and IL-6did indeed detect these antigens in zebra finch, we conducted Western blot studies.

Birds were collected and decapitated after an overdose of Isoflurane. The telencephalons were rapidly dissected quickly frozen in a dry ice methanol bath and placed at -80°C until use. Brain tissue was homogenized in Hepes buffer [10 mM Hepes, 10 mM KCl, 0.1 mM EDTA, and 200 μl of 10% IGEPAL/5 ml of buffer A, 1 μl of Protease inhibitor (Sigma-Aldrich, St. Louis, MO)] and centrifuged at 4°C at top speed (15,000-16,000 × g) for 30 min and placed on ice. Supernatant was immediately removed and placed in -20°C until use. 20 μl of protein from each sample was run on 4-12% Tris-HCl gels (Bio-Rad, Hercules, CA). Protein was transferred to a PVDF membrane (Bio-Rad, Hercules, CA). Membranes were blocked in 5% non-dairy milk for 3 hours and incubated overnight at 4°C in primary antibody at 1:1000. Goat anti-rabbit secondary (Cell Signaling Technology Inc, Danvers, MA) was applied at 1:3,000 and incubated at room temperature for 30 min. After a series of washes in Tris-buffered saline the reaction was visualized with an ECL Western Blotting Detection kit (Pierce, Rockford, IL) on Hyperfilm ECL (Amersham Biosciences, Piscataway, NJ).

### Experiment 1: Temporal Expression of IL-1β-like, IL-6-like, and Aromatase protein following injury

#### Injury

Subjects were anesthetized (0.03-0.05 ml/15 g of Nembutal (pentobarbital sodium salt from Sigma-Aldrich, St. Louis, MO, 25 mg/mL in a 20% propylene glycol and 5% ethanol solution) and positioned in a stereotaxic apparatus with the head angled at 45°. A bilateral craniotomy was created at 2-mm anterior to the pineal gland and 3-mm lateral to the midline. Injuries were targeted toward the entopallial nucleus 3-mm ventral to the brain surface [34] because it lacks constitutively expressed neuronal aromatase [5,7,32,35]. A 22 s Hamilton syringe (Hamilton Company, Reno, NV) was positioned at the surface of the brain and lowered to the target where it remained for 60 secs.

#### Immunohistochemistry (IHC)

Whole brain sections from adult birds were removed from skull and fixed in 5% acrolein at either 2,4,8, 12, 24, or 72 hrs following penetrating injury. Tissue was cut on a cryostat at 40 μm after being gel embedded and stored in cryoprotectant at -20C until use. Following standard immunohistochemistry protocols, free-floating sections were washed 6 × 10 min in 0.1 MPB, then tissue was treated with 10% sodium borohydride in 0.1 M PB. Tissue was then placed in 10% normal goat serum and incubated with primary antibody in 0.1 M PB with 0.3% Triton-X (Sigma- Aldrich, St. Louis, MO) for 48 h at 4C. Anti-zebra finch aromatase C-terminal (AZAC) was used at a concentration of 1:20,000, while antibodies for IL-1β-like and IL-6-like proteins were used at a concentration of 1:75,000. Tissue was then washed 10 × 6 min in 0.1 M PB with 0.1% Triton-X (PBT) and placed in biotinylated rabbit secondary antibody for 60 min (Jackson ImmunoResearch Laboratories, Inc., West Grove, PA), washed again 3 × 10 min in 0.1 M PBT and then placed in A/B solution (VectaStain, Burlingham, CA) for 60 min. After incubation and a series of washes in 0.1 M PBT and 0.175 M sodium acetate sections were then placed in a chromagen solution containing diaminobenzidine tetrahydrochloride (Sigma-Aldrich, St. Louis, MO), hydrogen peroxide, and Nickel sulfate (Sigma-Aldrich, St. Louis, MO) to visualize the reaction. Sections were mounted on slides and then coverslipped after serial dilutions in alcohol and zylene (Fisher Scientific, Pittsburgh, PA). IHC were preformed simultaneously on brains to control for any between-run differences in staining procedures. Representative micrographs were obtained from non-overlapping sections surrounding the injury tract and rendered using NIH Image J. An experimenter blind to the source of the photomicrographs counted the total number of labeled cells within the injured entopallium per 0.3 mm^2^. Data from images within individual injuries and subjects were averaged, and group means representing the number of labeled cells were computed across treatment [35].

#### Co-Expression of Glial/Microglia Markers and Aromatase/IL-1β-like and IL-6-like

To determine the nature of cells expressing either aromatase or IL-1β-like and IL-6-like we used double-label immunocytochemistry. Vimentin (Developmental studies Hybridoma Bank, Iowa City, IA) has been previously used as a glial marker in the avian brain [36]. Sections were washed 6 × 10 min in 0.1 MPB, then tissue was treated with 10% sodium borohydride in 0.1 M PB. Tissue was then placed in 10% normal goat serum and incubated in a primary antibody cocktail containing 1:2500 AZAC [32] and 1:750 anti-vimentin in 0.3% PBT (48 h, 4C). Tissue was then washed 10 × 6 min in 0.1 M PB with 0.1% Triton-X (PBT) to remove excess primary and then incubated in a secondary antibody cocktail containing 1:50 mouse-adsorbed, goat anti-rabbit cyanine-5 (CY-5) and 1:50 rabbit-adsorbed, goat anti-mouse cyanine-2 (CY-2; Jackson Immunochemicals, West Grove, PA) in 0.3% PBT for 2 h at room temperature under foil. Sections were then washed in 0.1% PBT for a total of 2 h, mounted, dehydrated, and coverslipped. For studies examining the co-expression microglial markers and cytokines, sections were washed 6 × 10 min in 0.1 MPB, then tissue was treated with 10% sodium borohydride in 0.1 M PB. Tissue was then placed in 10% normal goat serum and incubated in a primary antibody at 1:50,000 for 48 h at 4C. Tissue was then washed 10 × 6 min in 0.1 M PB with .1% Triton-X (PBT) to remove excess primary and then incubated in a secondary antibody cocktail containing 1:50 mouse-adsorbed, goat anti-rabbit cyanine-5 (CY-5) and 1:1000 isolectin GS-IB_4 _from *Griffonia simplicifolia*, Alexa Fluor^® ^488 conjugate (Invitrogen, Carlsbad, Ca) for 2 h at room temperature under foil. Sections were then washed in 0.1% PBT for a total of 1 h, mounted, dehydrated, and coverslipped. Isolectin B4 stains microglial cells in the adult CNS and also has a lower affinity to vascular endothelial cells [37,38].

#### Confocal Microscopy

Sections double-stained with antibodies against vimentin/aromatase or isolectin(IB4)/IL-1 or IL-6 were inspected under a scanning confocal microscope. Sections were observed using a 10X or 25X/1.4 NA plan apo objective on an inverted microscope (Zeiss Axiovert 200 M) equipped with a Zeiss LSM510 META scan head. Argon ion, 543 HeNe, and 633 HeNe lasers were used to generate the 488, 543, and 633 lines used for excitation, and pinholes were typically set to 1-1.5 airy units.

### Experiment 2: Phytohemagglutin (PHA) induced neuroinflammation on glial aromatase expression

#### PHA induced neuroinflammation

Subjects were anesthetized (0.03-0.05 ml/15 g of Nembutal (pentobarbital sodium salt from Sigma-Aldrich, St. Louis, MO, 25 mg/mL in a 20% propylene glycol and 5% ethanol solution) and positioned in a stereotaxic apparatus with the head angled at 45°. A bilateral craniotomy was created at 2-mm anterior to the pineal gland and 3-mm lateral to the midline. Either phytohemagglutin [PHA, stimulates T lymphocytes to release cytokines, 39] or saline was dripped over the open surface of the brain, with special care taken not to damage the brain or the dura mater.

#### Immunohistochemistry (IHC)

The entire brain was removed from the skull and fixed in 5% acrolein either 6 h or 24 h following neuroinflammation. Tissue was cut on a cryostat at 40 μm after being gel embedded and stored in cryoprotectant at -20C until use. The same protocol stated above was used for analysis of cytokines and aromatase. For analysis of cytokine immunoreactivity densitometry, at least four images were taken per animal and an uncalibrated optical density was calculated using NIH Image J and averaged across the images and animals. To determine the extent of programmed cell death induced by the penetrating injury vs. PHA induced neuroinflammation, sections were exposed to terminal deoxynucleotidyl transferse UTP nick end labeling (TUNEL), which labels the ends of degenerating DNA, thereby permitting visualization of apoptotic nuclei. The sections were processed as previously published [7] using the TUNEL Apoptosis Detection Kit (Upstate Biotechnology, Waltham, MA).

#### qPCR

Birds (n = 8) were decapitated 24 hours following treatment and each telencephalic lobe rapidly dissected out into 1 mL of Trizol reagent (Invitrogen, Carlsbad, CA, USA) and completely homogenized. Total RNA was isolated from the samples using the methods suggested by the manufacturer. Samples were analyzed on a ND-1000 spectrophotometer (NanoDrop, Wilmington, DE, USA), and only those samples that had a 260/280 ratio that exceeds 1.85 were used. For every qPCR experiment, 3 g of total RNA was reverse transcribed with an oligo(dT)_20 _using the Superscript III first strand synthesis kit for reverse transcription polymerase chain reaction (RT-PCR; Invitrogen, Carlsbad, CA, USA). For qPCR, 1 μL (or 5% of the total first strand synthesis reaction) of the resulting cDNA was amplified with Power SYBR Green PCR master mix (Applied Biosystems, University Park, IL, USA) in 25 μL of total reaction volume. Assays were done in 96-well optical plates and each sample was amplified in triplicate. In every run, wells without the RT product were included in order to detect any external contamination. Amplicons were generated against exons of the zebra finch aromatase transcript, as well as the housekeeping gene glyceraldehyde-3-phosphate dehydrogenase [GAPDH; see 40]. Primers were designed using zebra finch aromatase and GAPDH-specific sequences found in the GenBank database (GenBank accession numbers AF170274, AF170273, S75898 and AF255390).

#### Statistics

The mean cell counts for IL-1β-like, IL-6-like, and aromatase were analyzed separately using a one-way analysis of variance with treatment as the variable. These test were followed by Tukey-Kramer HSD post hoc analysis for pairwise comparisons when significant main effects were determined. mRNA expression data were analyzed using a t-test, with treatment as the main variable. Densitometry data were analyzed using an ANOVA with treatment and time following surgery as the main variables. Post hoc analysis was used to determine individual differences. JMP statistical analysis software (SAS, Cary, NC) was used to generate analysis. For all test results a predetermined level of α = 0.05 was used.

## Results

### Specificity of antibodies

**Figure 1:**
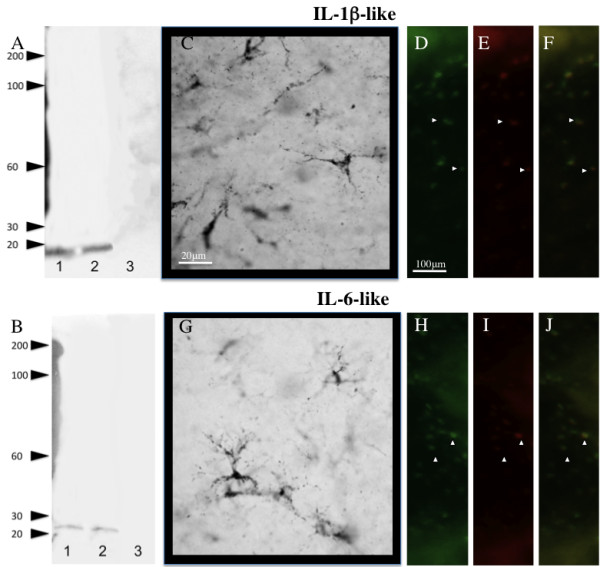
**Interleukin antibody specificity and cellular characterization**. Western blot analysis of IL-1β-like (A) and IL-6-like (B). Representative high power magnification of IL-1β-like (C) and IL-6-like (G) immunoreactive cells in the zebra finch brain. IL-1β-like (D-F) and IL-6-like (H-J) cells around injury coexpress microglial proteins (yellow, (F, J). Panels (D-E) and (H-I) reveal the identical field of cells viewed through the green (microglia) and red (IL-1β-like and IL-6-like) channels alone.

To the best of our knowledge, immunoreactive cytokines have not been described in the passerine brain. Consequently, IL-1β-like and IL-6-like proteins were detected using chicken (Gallus gallus) specific antibodies (Pierce, Thermo Scientific; Rockford, IL). The predicted zebra finch IL-1β-like and IL-6-like nucleotide and protein sequences had high sequence identity (> 80%) with chicken nucleotide and protein identities. These data indicate a high homology between the two avian species and suggest a high likelihood of specific antibody binding. Also to test for the specificity of these antibodies, western blot analysis was run. Western blot analysis revealed a single band of an apparent size of 17 kD detected for IL-1β-like and of 21 kD for IL-6-like (Figure 1A-B).

### Temporal Expression of IL-1β-like, IL-6-like, and Aromatase following injury

#### IL-1β-like immunoreactivity

**Figure 2:**
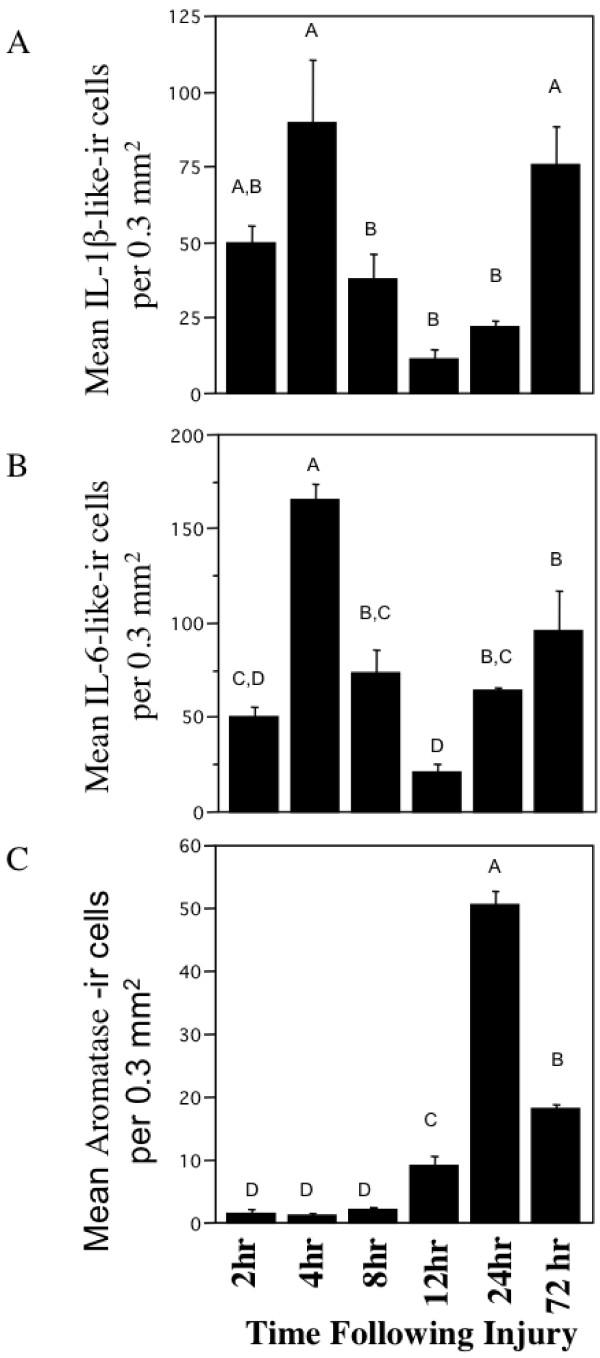
**Temporal expression of IL-1β-like immunoreactive cells (A), IL-6-like (B), and Aromatase (C) following mechanical injury in the zebra finch brain**. Data are represented as mean ± SEM. Data not connected by the same letter are significantly different (p < .05).

IL-1β-like immunoreactivity (IL-1β-like-ir) was detectable as early as 2 hrs post-injury, but was dramatically higher at 4 hrs where it reached maximum. IL-1β-like-ir declined following 4 hrs following injury, however began to increase again 24 hrs post injury and was significantly different across the times examined (Figure 2A; F_5,15_= 6.4938, p = 0.0061). Anatomical structural analysis and coexpression studies suggest that these cells are microglia due to their differences in shape and size when compared to both neuronal and astrocytic aromatase (Figure 1C-F).

#### IL-6-like Immunoreactivity

IL-6-like immunoreactivity (IL-6-like-ir) was also detectable at 2 hrs and was maximal at 4 hrs post-injury, and followed a similar pattern of expression as IL-1β-like-ir. Notably, IL-6-like-ir differed from IL-1β-like-ir 24 hrs following injury where IL-6-like-ir was back to levels observed at 8 hrs following injury, unlike IL-1β-like-ir which showed a slower increase in expression. Similar to IL-1β-like-ir, IL-6-like-ir was significantly different following injury (Figure 2B; F_5,15_= 16.7474, p = 0.0001). Anatomical structural analysis and coexpression studies suggest that these cells are microglia as well (Figure 1G-J).

#### Aromatase Immunoreactivity

Maximal expression of aromatase occurred at 24 hrs following a rise of expression that started 8 hrs following injury and dropped at 72 hrs following injury. Despite this drop in expression between 24 and 72 hrs, expression at 72 hrs was still higher than 0-12 hrs post injury where aromatase immunoreactivity is lowest, and was significantly different following mechanical injury (Figure 2C; F_5,15_= 355.5745, p < 0.001). Injury induced glial aromatase immunoreactive cells have been previously described as astrocytes and not microglia [1,41].

PHA induces a neuroinflammatory response in the zebra finch brain independent of a penetrating injury.

**Figure 3:**
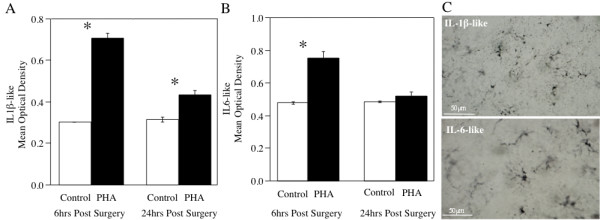
**Bar graph depicting the mean uncalibrated optical density (OD) for IL-1β-like (A) and IL-6-like (B) immunoreactive cells at 6 h and 24 h following treatment with PHA**. (C) Representative sections depicting the localization of IL-1-like (top) and IL-6-like -ir cells (bottom) following PHA treatment. * denotes a significant difference between treatment and saline.

To confirm that PHA treatment was truly related to neuroinflammation two markers of neuroinflammation were examined (IL-1β-like and IL-6-like; Figure 3). 6 h following surgery, both IL-1β-like and IL-6-like were upregulated over controls. Statistical analysis was used to confirm this effect and found that both IL-1β-like (Figure 3A) and IL-6-like (Figure 3B) were significantly different than controls (F_3,15 _= 110.73, p < 0.0001; F_3,15 _= 28.4698, p < 0.0001), respectively. This effect appears to be short term as cytokines appear to return to baseline 24 hours following treatment anatomical and structural analysis suggests that these immunoreactive cells are microglia as opposed to astrocytes (Figure 3C).

#### Aromatase expression following neuroinflammation

**Figure 4:**
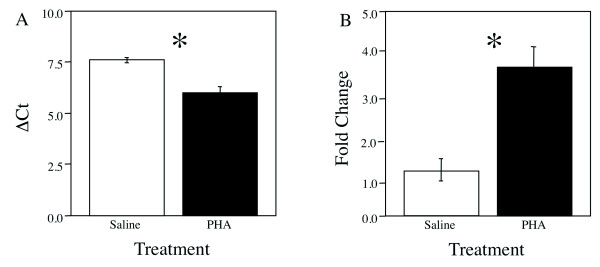
**Bar graph showing the mean ± SEM in ΔCt values (normalized against GAPDH, (A)) and fold change (B) between saline and PHA treated telencephalons for aromatase**. Fold change in expression was calculated using the double delta Ct method assuming 100% efficiency. * denotes a significant difference between treatment and saline.

Aromatase mRNA expression was greater on the side of the brain exposed to PHA than exposed to saline (Figure 4A). Comparative Ct measurements give a relative expression difference between samples, where a lower number means greater expression and were used to determine the mRNA expression following treatment. 24 h after inducing a proinflammatory response, the PHA groups had significantly lower delta Cts (increased mRNA expression) than saline controls (t(13) = 2.868, p < 0.0100; Figure 4A). Data was converted to fold-change and showed that there was significant difference between the control and PHA treated areas, with PHA treatment significantly increasing aromatase expression over control (t(13) = 4.857, p = 0.0003; Figure 4B).

#### Aromatase immunoreactivity following PHA treatment

**Figure 5:**
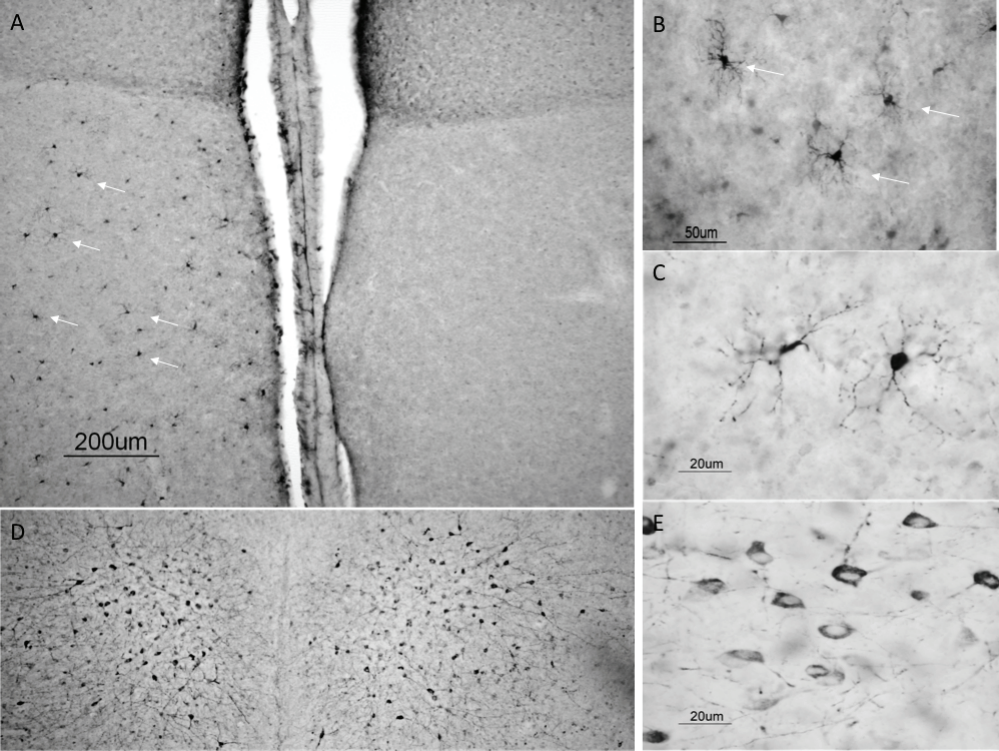
**Representative sections from male zebra finches for aromatase immunoreactivity (Arom-ir) following exposure to PHA and saline**. (A) Low power magnification of Arom-ir following exposure to PHA (left) and saline (right). (B) Higher power magnification of Arom-ir cells after exposure to PHA. (C) High power magnification exhibiting the typical cellular morphology and structure of Arom-ir cells following exposure to PHA. (D) Control section showing that normal Arom-ir is not affected by treatment with PHA (left) or saline (right). (E) High power magnification showing neuronal expression of aromatase.

**Figure 6:**
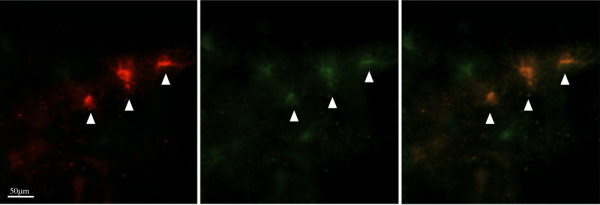
**Panels (A) and (B) reveal the identical field of cells viewed through the red (aromatase (B)) and green (vimentin (C)) channels alone**. Aromatase cells coexpress glial proteins (yellow, C).

PHA induced aromatase expression in glia without a penetrating injury. Exposing the brain to PHA, but not saline increased expression of aromatase ir-cells 24 h following surgery (Figure 5). Further analysis of aromatase expression suggests that the induction of glial aromatase is localized to the side of the brain that received PHA treatment only (Figure 5A) and that neuronal aromatase is present on both sides of the brain (Figure 5D-E). Also, the size and shape of aromatase ir-cells suggest that the cells present are in fact glia (Figure 5B-C) and coexpression with vimentin confirmed that these cells are indeed astrocytes (Figure 6).

#### Cell death Assay

**Figure 7:**
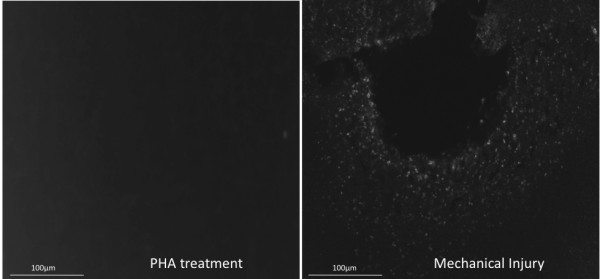
**Representative sections black-white inverted following cell degeneration assay (TUNEL)**. TUNEL- labeled cells were not present in tissue exposed to PHA (A). PHA tissue was run concurrently with injured tissue to serve as a positive control (B).

PHA induced neuroinflammation does not cause cell death. Following a penetrating injury increased cell death and increased glial aromatase is observed. In the PHA groups, we observed increased aromatase expression, but no corresponding increased cell death markers (Figure 7).

## Discussion

In this study, we examined the temporal pattern of IL-1β-like and IL-6-like and aromatase following injury to the zebra finch brain and whether neuroinflammation affects glial aromatase transcription and translation. Following injury both IL-1β-like and IL-6-like protein were localized around the injury site, with maximal expression occurring at 4 hrs post injury. In contrast aromatase expression increased about 24 hrs post injury. Thus, cytokine expression precedes aromatase expression and thus could serve as the biological inducer of glial aromatase expression. To further examine the role that cytokines may serve in inducing neuroendocrine factors such as aromatase we induced a neuroinflammatory state characterized by increased expression of two cytokines (IL-1β-like and IL-6-like) and was able to increase glial aromatase transcription and translation. Importantly, this neuroinflammatory state was not accompanied by cell death and occurred prior to the increase in glial aromatase transcription and translation. These data indicate that cytokines may serve to induce neuroendocrine factors such as aromatase, which in turn, is neuroprotective.

Prior to this study, a mechanism to explain the induction of glial aromatization in the avian and mammalian brain following injury or damage was yet forthcoming. Two major physiological events that accompany brain injury are; (a) disruption of the blood brain barrier with a consequent inflammatory response that helps and hinders repair and (b) cell death during and following the primary injury [21,22]. Subsequently, both could be plausible signals underlying glial aromatization.

### Role of neuroinflammation on glial aromatase expression

Expression of both IL-1 and IL-6 preceded aromatase expression and without any noticeable cell death we were able to induce glial aromatase expression with neuroinflammation alone. Studies in non-neuronal tissue (breast and ovarian) show that IL-1β and IL-6 upregulate aromatase expression [27,30,42-45]. The present data extend these findings into the brain *in vivo *and suggest a novel role for cytokines on the initiation of glial aromatase expression. Interestingly, estrogens (the expected result of increased glial aromatase expression) can also counteract neuroinflammation by targeting expression of proinflammatory cytokines from astrocytes [46,47]. Studies in the songbird have elucidated that glia-derived estrogen may act by decreasing reactive gliosis that inhibits neurodegeneration [11]. Both cytokines studied did decline, however this drop in expression occurred prior to a significant increase in aromatase expression. We have previously shown that aromatase expression is still upregulated 6 weeks following injury [11], and the lack in a decline in cytokine expression may be due to a temporal delay in the conversion of steroidogenic precursors to estrogen that may extend beyond the time-points studied. Also, aromatase via estrogens may mitigate a greater rise in cytokine response at later time-points (> 72 hrs). Selective estrogen receptor modulators (SERM) have been studied for their use and have proven to be just as useful as estradiol alone in decreasing cytokine expression and neuroinflammation [46,48,49]. Interestingly, we detected a decrease in cytokine production following PHA exposure, this decrease in inflammatory response may be due to transient nature of the inflammatory response itself or due to the predicted increase in estrogen production. Future research is needed to elucidate which mechanism is responsible for the decline in cytokine expression following PHA treatment. However, these data suggest that neuroprotection is mediated by the classic activation of both ER-α and/or ER-β that subsequently increase gene expression of factors regulating neuroprotective mechanisms [46,50,51].

### Role of cell death on glial aromatase expression

As stated previously, cell death is a consequence of primary injury to the brain, thus cell death both pyknotic and apoptotic could be a mechanism underlying the induction of glial aromatase. Therefore, we compared apoptosis levels in the PHA-exposed brain to that of a mechanically injured brain and failed to detect cell death or degeneration due to PHA alone. Since cell death was readily detectable due to mechanical injury the data suggest that neither PHA induced neuroinflammation nor the surgery to expose the brain to PHA result in cell death and thus cell death is not necessary to increase glial aromatase expression. However it is important to point out that estrogen provision is a potent mitigator of apoptotic secondary degeneration and both apoptosis and neural injury are markedly decreased by glial aromatase [7,11,15,35,52,53].

### Cytokines as neuroprotective

Presumably, PHA caused the release of cytokines from microglial following T-lymphocyte stimulation [39] following exposure to the neuropil. This release of cytokines then creates and inflammatory response and notably, can exacerbate neurodegeneration [17,19,21,54]. However recent evidence suggests that expression of cytokines can also be neuroprotective via expression of factors related to neurogenesis and repair [23,55,56]. Interestingly, a second rise in cytokine expression occurred in the injury tissue and may signal the shift from neurodegeneration to repair in damaged tissue. IL-6 in particular, is known to shift from a pro-inflammatory mediator to an anti-inflammatory/neuroprotective role following injury [57-59]. This shift also occurs in both the localization and function of IL-1β following injury. Initially of IL-1β is localized within microglia and serves as a pro-inflammatory cytokine, however later the expression of IL-1β can shift to astrocytes and serves to increase the production of inflammatory mediators [60-63]. Further research is needed to fully test this hypothesis, however the rise in aromatase expression may serve to also shift the expression and localization of these cytokines into a neuroprotective state and thus prolong the expression of glial aromatase.

### Neuroimmune-Neuroendocrine Interactions as a novel mechanism of neuroprotection

**Figure 8:**
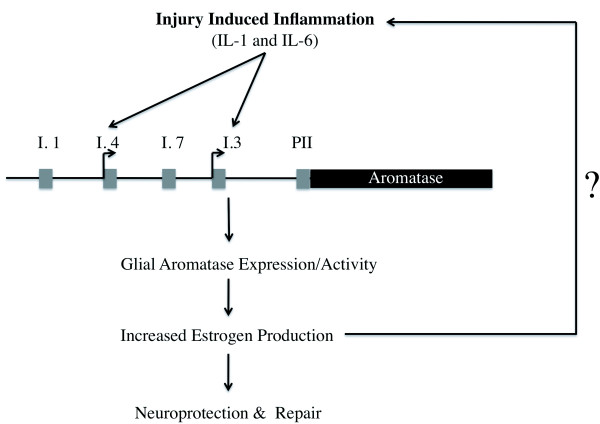
**Proposed model of aromatase mediated neuroprotection following injury or damage to the brain**.

We have presented an interaction between cytokines and glial aromatase and thereby neurodegeneration, and neuroprotection. These data along with our own, provide a conceptual framework for the mechanisms underlying cytokines, aromatase, and estrogens in the brain following injury (Figure 8). These data support the emerging theory that neuroinflammation and cytokine expression following injury are not always detrimental to the organism, but may be necessary for the neuroprotective actions of aromatase and estrogen [23,55,56]. While cytokines appear to increase neuroinflammation, which can be very damaging to the brain, they also activate genes to protect the brain against further damage. These protective genes may also have a reciprocal action that decrease the cytokine response quite possibly limiting the extent of the neuroinflammation as well. Further research is needed to fully understand this complex interaction between neurotoxicity and neuroprotection.

## Conclusions

We show that the cytokine response occurs prior to the glial aromatase response, and strongly suggest that cytokine expression is necessary for the shift in purely neuronal aromatase expression to a population of both neuronal and glial aromatase. Our findings suggest that following TBI, increased inflammation caused by increased cytokine expression evokes a neuroprotective pathway involving aromatase. This increased aromatase expression may represent an interesting candidate to counteract neuroinflammation under times of stress. Studies using the songbird as a model have revealed a unique and endogenous protective mechanism that reduces neurodegeneration and may actively inhibit the deleterious effects of prolonged cytokine action following brain damage. These data are key to our understanding of the mechanisms regulating aromatase-mediated neuroprotection.

## Competing interests

The authors declare that they have no competing interests.

## Authors' contributions

KAD participated in the design of the study, carried out the molecular and immunhistological studies, and performed the statistical analysis, and helped to draft the manuscript. CJS participated in its design and coordination and helped to draft the manuscript. All authors read and approved the final manuscript.
